# Identification of a spliced gene from duck enteritis virus encoding a protein homologous to UL15 of herpes simplex virus 1

**DOI:** 10.1186/1743-422X-8-156

**Published:** 2011-04-06

**Authors:** Hongwei Zhu, Huixin Li, Zongxi Han, Yuhao Shao, Yu Wang, Xiangang Kong

**Affiliations:** 1 Division of Avian Infectious Diseases, State Key Laboratory of Veterinary Biotechnology, Harbin Veterinary Research Institute, the Chinese Academy of Agricultural Sciences, Harbin 150001, China

## Abstract

**Background:**

In herpesviruses, UL15 homologue is a subunit of terminase complex responsible for cleavage and packaging of the viral genome into pre-assembled capsids. However, for duck enteritis virus (DEV), the causative agent of duck viral enteritis (DVE), the genomic sequence was not completely determined until most recently. There is limited information of this putative spliced gene and its encoding protein.

**Results:**

DEV UL15 consists of two exons with a 3.5 kilobases (kb) inron and transcribes into two transcripts: the full-length UL15 and an N-terminally truncated UL15.5. The 2.9 kb UL15 transcript encodes a protein of 739 amino acids with an approximate molecular mass of 82 kiloDaltons (kDa), whereas the UL15.5 transcript is 1.3 kb in length, containing a putative 888 base pairs (bp) ORF that encodes a 32 kDa product. We also demonstrated that UL15 gene belonged to the late kinetic class as its expression was sensitive to cycloheximide and phosphonoacetic acid. UL15 is highly conserved within the *Herpesviridae*, and contains Walker A and B motifs homologous to the catalytic subunit of the bacteriophage terminase as revealed by sequence analysis. Phylogenetic tree constructed with the amino acid sequences of 23 herpesvirus UL15 homologues suggests a close relationship of DEV to the *Mardivirus *genus within the *Alphaherpesvirinae*. Further, the UL15 and UL15.5 proteins can be detected in the infected cell lysate but not in the sucrose density gradient-purified virion when reacting with the antiserum against UL15. Within the CEF cells, the UL15 and/or UL15.5 localize(s) in the cytoplasm at 6 h post infection (h p. i.) and mainly in the nucleus at 12 h p. i. and at 24 h p. i., while accumulate(s) in the cytoplasm in the absence of any other viral protein.

**Conclusions:**

DEV UL15 is a spliced gene that encodes two products encoded by 2.9 and 1.3 kb transcripts respectively. The UL15 is expressed late during infection. The coding sequences of DEV UL15 are very similar to those of alphaherpesviruses and most similar to the genus *Mardivirus*. The UL15 and/or UL15.5 accumulate(s) in the cytoplasm during early times post-infection and then are translocated to the nucleus at late times.

## Background

Duck enteritis virus (DEV), also known as Anatid herpesvirus-1 (AHV-1), is an important pathogen of birds of the order *Anseriformes*, including ducks, geese and swans, causing the acute contagious disease duck viral enteritis (DVE) or duck plague (DP), which results in substantial mortality and reduction of egg production in domestic as well as in wild waterfowl [1,2]. DEV was classified as an unassigned virus within the family *Herpesviridae *according to the Eighth International Committee on Taxonomy of Viruses (ICTV) [3]. Evidence from recent phylogenetic analysis of the nucleotide sequence or the predicted amino acid sequence suggests that DEV was closely related to the genus *Mardivirus *or *Varicellovirus *and might represent a single cluster within the subfamily *Alphaherpesvirinae *[4-8].

The genomic sequence of DEV was determined and analyzed recently and the presence of more than 78 different open reading frames (ORFs) was predicted [8]. A terminase-related, protein-encoding gene homologous to HSV UL15 was predicted based on the homology analysis of theses ORFs and those of their homologous counterparts from other herpesviruses. The UL15 is a rarely occurring spliced gene in herpesviruses and consists mostly of two exons [9-15]; however, there are three exons in UL15 homologue from the channel catfish herpesvirus [16]. UL15 is highly conserved among members of the family *Herpesviridae*, which implies the functional significance of this protein [9]. The amino acid sequence of UL15 shares homology with the large subunit of the terminase complex of bacteriophage T4, particularly with respect to the two nucleotide-binding motifs of the ATP-binding domain known as Walker A and Walker B domains [16,17], which suggests UL15 is ATP binding as demonstrated for gp17 and other phage terminases [18].

Within the infected cells, UL15 and at least other six proteins (UL6, UL17, UL25, UL28, UL32 and UL33) are involved in viral DNA cleavage and packaging [19-25]. In accordance with the defined role as a terminase subunit, the UL15 protein is presumed to localize or colocalize with other subunits primarily within the nucleus of infected cells. Indeed, HSV-1 UL15 is transported from intranuclear space at 6 h post-infection (h p. i.) into the nucleus at 12 h p. i. and localizes to the replication compartments where cleavage and packaging proteins might be recruited to process the viral DNA, as indicated by immunofluorescence assay [21,26]. In addition, the ORF45/42 gene product, a UL15 homologue in varicella-zoster virus (VZV), also shows nearly exclusive nuclear localization in infected cells [14]. In contrast to its HSV-1 homologue, cells transiently transfected with ORF45/42 showed only weak nuclear staining, which suggests the formation of a protein complex, or at least a heterodimers, by UL15 homologues and other putative terminase subunit(s). Such a terminase complex has been studied extensively in HSV-1 [27-29] and in human cytomegalovirus (HCMV) [30].

In DEV, the UL15 transcript feature and its encoding product were analyzed merely on the basis of the sequence alignment with its counterparts from other herpesviruses. Little is known about this, or at least possible, spliced gene and its product in DEV-infected cells or DEV virion. Therefore, we describe the identification and characterization of the putative UL15 transcript and its encoding protein in DEV-infected cells, which provides data to aid the classification of DEV within the *Herpesviridae *family and provides clues for better understanding the possible role of UL15 in DEV infection.

## Results

### UL15 is a spliced gene with two transcripts

**Figure 1:**
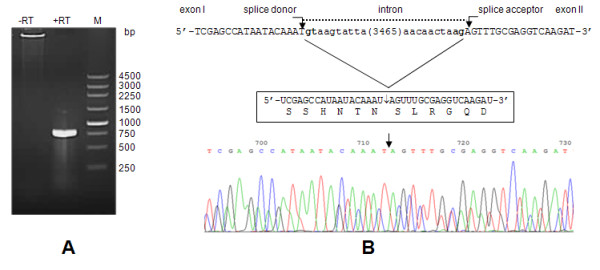
**The result of splice junction mapping**. (A) The RT-PCR product obtained with primers Psjf and Psjr in DEV-infected CEF cells. The primers were expected to yield a fragment of 786 bp as the spliced UL15 product. DNA marker fragments are shown at the right (in bp). (B) Splice sites within the UL15 gene. Intron/exon boundaries are in bold and the splice sites are indicated by vertical arrows. Exon sequences are in uppercase and intron sequences are in lowercase. A number within brackets in the intron sequence is the number of nucleotides omitted. The mature mRNA fragment near the splice site and the corresponding amino acid residues are shown in the box and the sequencing result of the RT-PCR product is indicated below.

Nucleotide sequence of the putative DEV UL15 gene was determined, annotated and deposited in GenBank (accession numbers EF524094 and EF203707) previously by our laboratory. The UL15 gene of DEV is assumed to consist of two coding exons separated by an intron that contains ORFs UL16 and UL17 transcribed in the orientation opposite to that of UL15 [8]. To confirm this assumption, RT-PCR was carried out with RNA isolated from DEV-infected CEF cells with specific primers covering the putative splice site. As expected, a 786 bp DNA product was observed by electrophoresis in agarose gel (Figure 1A). The fragment was cloned and sequenced, and the splice site was located. The genomic sequences at the exon-intron boundaries were found to read 5'- GCATTGACAGgtacttgtct-3' and 5'-aacaactaagAGTTTGCGAG-3' (intron sequences are indicated in lowercase letters) as donor and splicing acceptor sequences respectively (Figure 1B), conforming to the consensus of the GT-AG rule [31].

**Figure 2:**
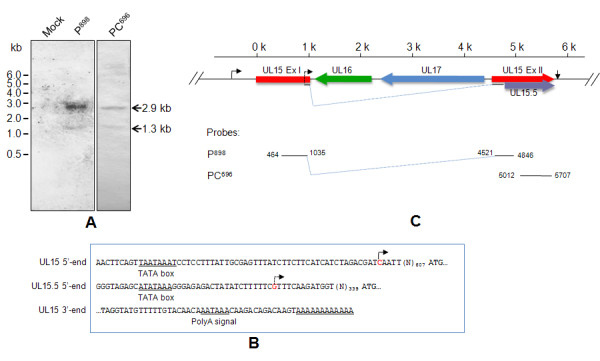
**Schematic diagram of the UL15 gene**. (A) Northern blot hybridization. Total RNA was isolated from DEV-infected CEF cells at 48 h p. i. RNA samples were then separated, blotted and hybridized with the probes and analyzed. The size of the mRNA was calculated according to the RNA size standard shown at the left. (B) The cDNA ends of UL15 and UL15.5. The promoters were predicted on the Berkeley *Drosophila *Genome Project's neural network promoter search engine (http://www.fruitfly.org/seq_tools/promoter.html). The TATA box and poly (A) signal elements were predicted on the TRANSFAC motif library (http://motif.genome.jp/) and the POLYADQ polyadenylation (polyA) signal search engine (http://rulai.cshl.org/tools/polyadq/polyadq_form.html), respectively. The predicted promoter sequences of UL15 and UL15.5 are shown, the TATA box, polyA signal motifs and polyA sequences are underlined, and the TSSs indicated by 5'-RACE are shown in red. The putative start and stop codons are in bold. (C) The DEV UL15 gene consists of two exons, exon I (Ex I) and exon II (Ex II), which are indicated by red arrows. The putative UL16 (green) and UL17 (blue) are located in the opposite orientation within the intron of UL15. The UL15 transcription start sites (TSS) and poly (A) sequences are indicated by black arrows. The UL15.5 TSS is indicated by the dashed arrow within Ex I of UL15. The ruler above the locus shows the relative position of the genes in kb (K).

The UL15 transcript was determined by northern blot analysis of total RNA isolated from mock-infected or DEV-infected cells. The results demonstrated that UL15 is transcribed with a predominant transcript of 2.9 kb and a less abundant transcript of 1.3 kb when hybridizing the blot with probes P^898 ^and PC^696 ^(Figure 2A). We believed that the 2.9 kb transcript corresponded in size to the UL15 transcript, while the 1.3 kb transcript corresponded in size to the predicted UL15.5 transcript.

### Sequence analysis of DEV UL15

The complete nucleotide sequence of the UL15 transcript is 2882 bp in length according to the 5'- and 3'-RACE, and the putative coding region is 2220 bp in length, encoding a 739 amino acid protein with a calculated molecular mass of 82.1 kDa. The UL15.5 transcript is 1290 bp in length with an 888 bp ORF that encodes a 32.1 kDa protein. The transcription start site (TSS) of UL15 is 612 bp upstream from the putative start codon ATG and that of UL15.5 is 352 bp upstream from the ATG codon. Promoters as well as TATA box elements of UL15 and UL15.5 were predicted at the 5'-cDNA end (Figure 2B). In addition, a consensus polyadenylation site (5'-AATAAA-3') was present 18 bp downstream from the UL15 stop codon. 14 bp downstream, this signal is 12 adenine residues, as indicated by 3'-RACE sequencing (Figure 2B); this polyadenylation site could define the 3' ends of both the UL15 and UL15.5 transcripts. The UL15 gene locus was thus drawn accordingly (Figure 2C).

**Figure 3:**
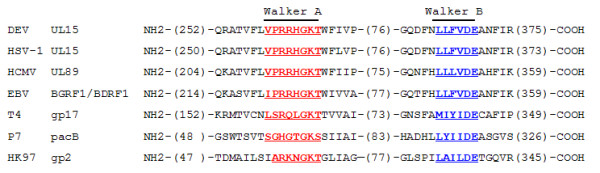
**Alignment of potential ATPase domains of the large terminase subunit from herpesviruses and bacteriophages**. Large terminase sequences of DEV(UniProtKB accession code C6ZD26), HSV-1 (P04295), HCMV (P16732), EBV (P03219), bacteriophage T4 (P17312), bacteriophage P7 (Q5XLR0) and bacteriophage HK97 (Q9MCT1) were aligned with the ClastalW2 multiple sequence alignment program[42]. The P7 terminase sequence was adjusted manually for the significant deviation in the distance between the motifs. The putative Walker A motifs of each sequence are underlined and in red and the Walker B motif are in blue. Numbers in brackets are the number of residues omitted.

As shown in Table 1, homologues of the DEV UL15 gene product are conserved among subfamilies of the *Herpesviridae*. UL15 shares a high level of amino acid sequence identity (32.9 - 62.3%) and similarity (50.3 - 75.2%) with nine other typical herpesviruses, as indicated by sequence alignments. Furthermore, two putative nucleotide-binding motifs, resembling the Walker A motif (GXXGXGKT/S, where X is any amino acid) and the Walker B motif (ZZZZD or ZZZZDE, where Z is a hydrophobic amino acid) exist within the UL15 ORF. These two characteristic motifs were found to read _261_VPRRHGKT_267 _and _354_LLFVDE_359_, and fit neatly with those from typical subfamilies *Alphaherpesvirinae *(HSV-1), *Betaherpesvirinae *(HCMV) and *Gammaherpesvirinae *(EBV), bacteriophage T4, bacteriophage P7 and bacteriophage HK97 (Figure 3).

**Table 1:** Amino acid sequence similarity of DEV UL15 to terminase homologs from other nine herpesviruses

Viruses	UL15 homologues	Identity percentage	Similarity percentage
HSV-1	UL15	59.6	74.9
HVT	UL15	62.3	75.2
MDV-1	UL15	58.2	73.4
VZV	ORF42/45	56.0	72.9
ILTV	UL15	46.4	61.9
PRV	UL15	52.8	64.7
HCMV	UL89	36.9	52.2
MCMV	M89	36.5	53.8
EBV	BGRF1/BDRF1	32.9	50.3

**Figure 4:**
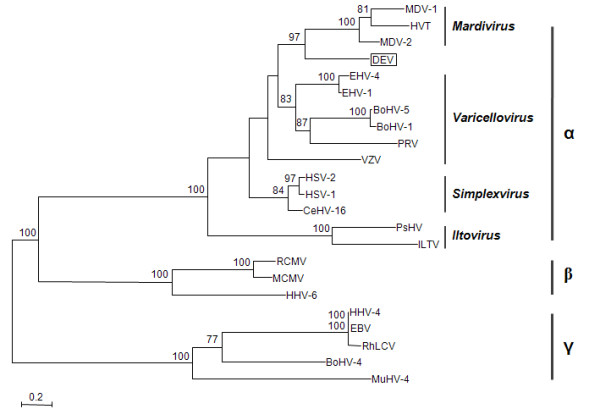
**Phylogenetic tree based on the UL15 protein sequences of 23 herpesviruses**. Amino acid sequences of UL15 homologues from 23 herpesviruses were retrieved and aligned using the ClustalW2 program. An unrooted phylogram was constructed using the PhyML software (version 3.0) with the LG amino acid substitution model and the non-parametric bootstrap support test. SeaView 4.1 was used for displaying and editing phylogenetic trees [43]. Values of bootstrap supports (100 replicates) in excess of 70% are shown on the shoulder of branches. The scale bar indicates 0.1 substitution/site and vertical lines are used to represent different subfamilies within the Herpesviridae and genera within the Alphaherpesvirinae.

Result from phylogenetic tree based on UL15 sequences of 23 herpesviruses showed that the DEV and other members in the *Alphaherpesvirinae *are clustered within a monophyletic clade with 100% bootstrap. Although the branch pattern of DEV UL15 shows distinctive character, its phylogenetic relationship in alphaherpesviruses, however, is still closely related to *Mardivirus *(Figure 4).

### UL15 is expressed late during infection

**Figure 5:**
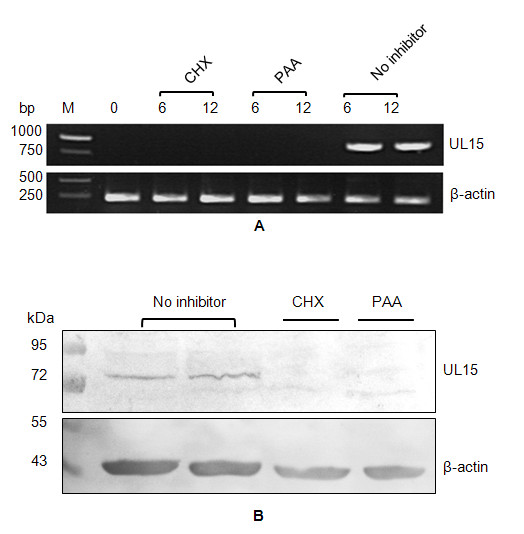
**Kinetic class analysis of DEV UL15**. DEV-infected CEF cells were harvested at the indicated time post-infection, followed by mRNA detection by RT-PCR (A) or protein detection by western blot (B). The procedures were done with 100 μg/ml cycloheximide (CHX) or with 300 μg/ml phosphonoacetic acid (PAA) or without metabolic inhibitor as the control. Chicken β-actin mRNA or protein was used as an internal control.

To determine which kinetic class UL15 gene belongs to, RT-PCR was performed to detect UL15 and chicken β-actin mRNAs respectively in the presence of CHX or PAA. The result showed that the UL15 mRNA was not detected in DEV-infected cells in the presence of CHX or PAA, while the RT-PCR product could be amplified in the absence of these metabolic inhibitors; as a control, β-actin mRNA was insensitive to CHX or PAA (Figure 5A). Similar to the finding from RT-PCR, the UL15-specific immunoreactive bands were detected only in the absence of drug inhibitors, as shown by western blot (Figure 5B). Taken as a whole, treatment with CHX or PAA inhibited transcription and expression of UL15 in infected cells, suggesting that the UL15 gene is transcribed and expressed as a late gene during DEV infection in CEF cells.

### Detection of UL15 in infected cells and DEV virion

**Figure 6:**
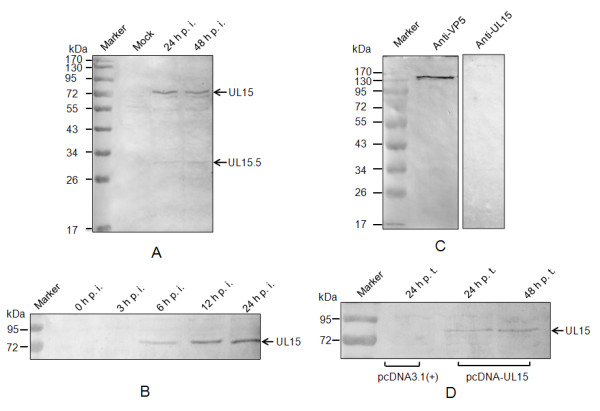
**Western blotting analysis of UL15 in infected cells and purified DEV virion**. (A) Mock or DEV-infected cells were harvested at the indicated time post-infection, separated by SDS-PAGE and analyzed by western blotting using antiserum to UL15. The arrows at the right indicate UL15 and UL15.5. (B) Time-course of UL54 accumulation during DEV replication. (C) The purified DEV virion was separated by SDS-PAGE, blotted and immunodetected using UL15 antiserum, and McAb AB5 against DEV VP5 as a positive control. (D) pcDNA-UL15 or pcDNA3.1 (+)-transfected cells were harvested at the indicated time post transfection, and analyzed by western blotting using antiserum to UL15. The positions of molecular mass markers (in kDa) are indicated at the left.

To characterize the expression of the UL15 gene in infected cells, western blot was performed on the DEV infected or pcDNA-UL15 transfected CEF cell lysates using the UL15-specific antiserum developed in rabbits. We found that the antiserum could react with an 82 kDa protein in DEV-infected CEF cell lysates, but not in uninfected cell extracts (Figure 6A). The protein was detectable at 6 h p. i. and the reactive band became stronger with time up to 24 h p. i. (Figure 6B). Further, the antiserum could react, although weakly, with a second polypeptide with molecular mass of 32 kDa (Figure 6A), confirming the expression of the 1.3 kb transcript indicated by northern blot. We believe that this minor protein represents the UL15.5 of DEV. We also examined the DEV virion for its reactivity with anti-UL15 antiserum. As shown in Figure 6C, we were unable to detect UL15 in the prepared DEV virions, whereas the major capsid protein VP5 was detectable when reacting with McAb 1B5. In addition, transfection of the pcDNA-UL15 plasmid into CEF could also result in the expression of UL15 (Figure 6D).

### Subcellular localization of the UL15 protein

**Figure 7:**
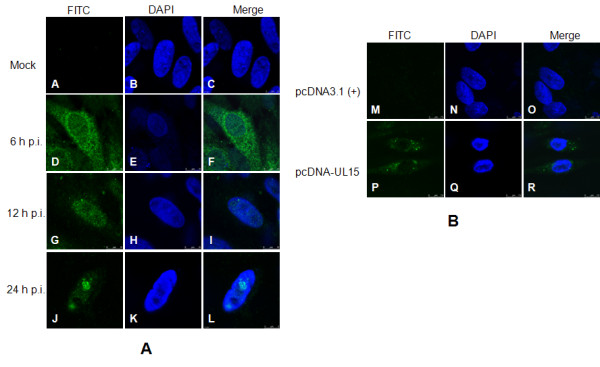
**Subcellular localization of DEV UL15 and/or UL15.5**. CEF cells were seeded onto Fluorodishes (WPI, Sarasota, FL, USA) and were infected with DEV or transfected with pcDNA-UL15. At the indicated time post-infection, cells were fixed with 4% paraformaldehyde (Sigma-Aldrich), followed by permeabilizing with 0.5% Trixon X-100. Immunofluorescence analysis was performed by laser scanning confocal microscopy using the UL15 antiserum and FITC-conjugated secondary antibodies. Nuclear DNA was stained in all cases with DAPI. Columns in the up-down direction show the individual FITC (left) and DAPI (middle) images. Merged visualization of these the images are shown in the right-hand column.

Subcellular distribution of DEV UL15 was determined by immunofluorescence assay of the DEV-infected CEF cells with anti-UL15 serum. As shown in Figure 7A, the fluorescence signals accumulated in the cytoplasm, predominantly in the perinuclear region of the cells in the early infection stage at 6 h p. i. At 12 h p. i. and 24 h p. i., however, the fluoresence signals localized mainly at the nucleus within the infected cells. Interestingly, at 24 h p. i., aggregation of fluorescence was observed occasionally within the nucleus (J and L in Figure 7), which might represent the so-called replication compartments where viral DNA synthesis occurs, which has been observed in HSV-1 [32]. In addition, specific fluorescence was limited to the cytoplasm rather than in the nucleus in pcDNA-UL15 transfected CEF cells (Figure 7B), which may indicated that the UL15 and/or UL15.5 protein(s) could not transport through the nuclear pore, or possibly that the protein(s) retained in the cytoplasm as a result of interacting with certain cellular protein(s). Specific fluorescence signal was not detected in any assay in the mock-infected cells or pcDNA3.1 (+) transfected cells.

## Discussion

It is common that eukaryotic DNA viruses encode genes containing introns that require mRNA splicing. However, the vast majority of transcripts are not spliced in viruses from the *Herpesviridae*. To our knowledge, aside from DEV UL15, there are four spliced UL15 homologues have been experimentally characterized in HSV-1, VZV, RCMV, Tupaia herpesvirus (THV) and Bovine herpesvirus 4 (BoHV-4) until now [9-12,14]. Remarkably in these five gene locus and other predicted homologues, we have noticed that although the coding sequences of UL15 homologues are conserved among herpesviruses, the ORFs and their arrangements between the exons are somewhat complex. In HSV-1, VZV and DEV, the intron contains two ORFs, UL16 and UL17, which have an orientation opposite to that of the UL15 gene [9,14]; in RCMV, the region between R89 (a UL15 homologue) exon 1 and exon 2 was found to contain five additional genes, r90, R91, R92, R93 and R94, the latter four are transcribed in the opposite orientation; in BoHV-4, the intron carries ORFs 30 - 33 in the opposite direction to UL15 homologues. It seems likely that the feature of gene arrangements within the introns is subfamily-specific within the *Herpesviridae*. This phenomenon might be the result of an evolutionary event: the terminase-related UL15 coding sequence remained conserved for its importance in herpesvirus biology, while genes within the intron evolved via recombination and gene transfer between virus and vertebrate hosts since these events are common in herpesviruses [33]. In this interpretation, the fact that the pattern of ORF organization within the DEV UL15 intron is very similar to that of the corresponding regions of other alphaherpesviruses is another piece of direct evidence that DEV is a member of the subfamily *Alphaherpesvirinae*, which is in accordance with pervious findings [4,6,7,34].

Two transcripts were detected using three different DIG-labeled DNA probes derived from the UL15 RT-PCR product as indicated by northern blot (Figure 2A). Although choosing probes from the putative UL15 ORF could not exclude the possibility that either detected transcript was the result of overlapping transcripts adjacent to the UL15 gene or transcribing from the UL15 intron, subsequent results from full-length cDNA mapping by RACEs showed that both transcripts were most likely expressed by UL15, which is in good agreement with the two polypeptides detected by the UL15 antiserum. Thus, we believe that the 2.9 kb and 1.3 kb transcripts are analogous to the UL15 and UL15.5 of HSV-1, respectively. Besides, the 1.3 kb transcript hybridized with the probes PC^696^, which might suggest the C-terminal location of the UL15.5 transcript. Furthermore, pairwise alignment of DEV UL15 with HSV UL15.5 suggested the 295 residues of the DEV UL15.5 coding sequence, with its translation initiation site, might be located at Met^445 ^within the DEV UL15 exon II. However, considering this methionine is not absolutely conserved among the herpesviruses family [16], whether this methionine is indeed an initiation site or translation of UL15.5 initiates at another site(s) requires further study. Interestingly, another four methionine residues (Met^449^, Met^455^, Met^457 ^and Met^462^) are present at this region only 4-17 residues downstream Met^445^, it is possible that when this site is mutated, another specific methionine might be utilized to translate a migrating version of UL15.5. In HSV-1, UL15.5 is dispensable for viral growth in the infected cell and its specific function during infection remains unknown, though several possible explanations for its origin and role have been discussed [26]. As for DEV UL15.5, further study is required to elucidate whether this gene is essential for DEV infection *in vitro *and *in vivo*, which will be a hint to specify the role(s) of UL15.5 itself as well as the C terminus of exon II concerning the largely identical sequences of the two.

In herpesviruses, there are several viral proteins participating in processing and packaging of viral DNA, among which UL15 homologue is a presumed terminase subunit with a potential role in the cleavage of progeny DNA [35]. The presence of the Walker A and Walker B box motifs in DEV UL15 might imply its corresponding role considering the following two facts: first, these two motifs fit well with the well characterized catalytic subunit (gp17) of the bacteriophage T4 terminase (Figure 3), similar position and distance between the motifs will ensure the correct formation of the nucleotide-binding site of an ABC (ATP-binding cassette) domain. Second, the role of the Walker A motif in viral genomic DNA cleavage in herpesvirus had been suggested by Przech et al., They found that HSV-1 point-mutated in the Walker A motif within the UL15 exon I failed to cleave viral DNA concatemers and the HSV-1 mutant exhibited growth defects [35].

To date, the taxonomic status of DEV is not clearly defined within the *Herpesviridae *in ICTVdb [3], although considerable evidence suggests it is a novel member or a member of the *Mardivirus *genus within the *Alphaherpesvirinae *[4,7,8]. As a genome-wide function-conserved protein among viruses within the *Herpesviridae*, the DNA packaging-related UL15 is believed to be inherited from a common ancestor by the Alpha-, Beta- and Gamma-herpesviruses [33,36]. This feature makes UL15 an ideal molecular genetic marker for phylogenetic construction and taxonomy within the family. For this reason, the UL15 protein has been widely chosen as one of the markers to characterize the evolutionary relationship of a certain virus within the family *Herpesviridae *[37-39]. As indicated from the tree based on deduced UL15 amino acid sequence, it is preferable to group the DEV as a member of *Mardivirus *genus, which is in accordance with the majority of the earlier findings [4,6,7].

As a component of terminase, UL15 is presumed to interact with replicating DNA and preassembled capsids to cleave and encapsidate the viral DNA. Thus, there exists at least a transient association of DEV UL15 with the capsids. However, UL15 was not detected in the purified DEV virion by western blot (Figure 6C). Two possibilities might account for the failure of UL15 detection. One is that UL15 is not a component of the DEV virion. The other is that it represents a virion component in a so low abundance that it is undetectable by the method used in this study. We would like to infer that the protein is present in the intranuclear capsid precursor however diminished in the mature virion. Theoretically, a mixture of procapsids and A-, B- and C-capsids are present in the DEV virion we have prepared, among which the C-capsid is the predominant type. Furthermore, only a trace amount of UL15 is present in procapsids, A- and B-capsids, whereas the protein might not be present in the more mature DNA-containing C-capsids, as demonstrated in its counterpart HSV-1 [40,41]. Therefore, it is possible that UL15 exists in the sucrose density gradient-purified virion, but at such a low abundance that it is not detectable by western blot. Analysis of the virion by more sophisticated techniques, such as immunoelectron microscopy, is needed to confirm this assumption.

Cytoplasmic nuclear transport of UL15 and/or UL15.5 was observed within the DEV-infected cells, which is consistent with the nuclear localization of the protein homologues observed in HSV-1 and VZV-infected cells [14,21]. These findings are in line with the fact that the UL15 homologue is a potential terminase subunit since viral DNA cleavage and encapsidation also take place within the nucleus. Interestingly, in HSV-1, UL15 expressed alone displayed efficient nuclear localization [26]; however, findings from VZV ORF45/42 transiently transfected cells as well as those from DEV UL15 in this study suggest that the protein is unable to localize to the nucleus in the absence of any other viral protein. This is not surprising as the UL15 ORF of HSV-1 contains a predicted short peptide (_183_PPKKRAKV_190_) resembling the reported nuclear localization signal (NLS) [21], whereas no similar NLS is predicted in either DEV UL15 or VZV ORF45/42 protein. Besides, ORF 29a/29b, another UL15 homologue from BoHV-4 does not contain a similar NLS sequence either. Hence, we tend to assume that the newly synthesized UL15 is localized in the cytoplasm due to the lack of NLS in DEV-infected cells, whereas transport into the nucleus when coupled with other viral and/or cellular protein(s) that contain NLS to form a protein complex, allowing the nucleus targeting of the protein. Further studies are needed to determine the protein(s) required for the cytoplasmic-nuclear trafficking of UL15 within DEV infected cells.

## Conclusions

DEV UL15 is a spliced gene that encodes two products encoded by 2.9 and 1.3 kb transcripts respectively. The UL15 is expressed late during infection. The DEV UL15 coding sequences are very similar to those of alphaherpesviruses and most similar to the genus *Mardivirus*. The UL15 and or UL15.5 accumulate(s) in the cytoplasm during early times post-infection and then are translocated to the nucleus at late times.

## Methods

### Cell culture, virus propagation and purification

Chicken embryo fibroblast (CEF) cells were prepared and maintained in Dulbecco's minimum essential medium (DMEM; Sigma-Aldrich, MA, USA) supplemented with 8% fetal bovine serum (FBS; JRH Biosciences, KS, USA), 2 mM l-glutamine, 100 units/ml penicillin and 100 μg/ml streptomycin. Primary or secondary cells were used to propagate DEV Clone-03, a CEF-adapted vaccine strain [6]. Cells infected with DEV were maintained in DMEM containing 2% FBS. To prepare the DEV virion, CEF monolayer was infected with DEV Clone-03 at a multiplicity of infection (m. o. i.) of 10 plaque-forming units (p. f. u.)/cell. Cells and the DEV-containing medium were harvested when more than 80% CPE developed, followed by 3 cycles of rapid freezing and thawing. The suspension was centrifuged at 4°C for 30 min at 3,000 × *g*, 30 min at 5,000 × *g *and 30 min at 8,000 × *g *to remove cell debris. The supernatant was recovered and centrifuged at 4°C for 2 h at 140,000 × *g*. The pellet was suspended in PBS and overlaid onto a discontinuous sucrose density gradient (20% and 60% sucrose in PBS) and centrifuged at 4°C for 2.5 h at 140,000 × *g *in a Beckman Coulter Optima™ L-100XP ultracentrifuge equipped with a SW41Ti swinging-bucket rotor (Beckman-Coulter, CA, USA). The virus band was collected and diluted with PBS. The virion pellets were finally collected after centrifugation at 4°C for 2 h at 140,000 × *g *and suspended in PBS.

### Reverse transcription polymerase chain reaction (RT-PCR)

CEF cells were infected with DEV Clone-03 at an m. o. i. of 10. Total RNA was extracted from the infected cells at 48 h p. i. with TRIzol^® ^reagent (Invitrogen, CA, USA) according to the manufacturer's instruction, followed by reverse transcription with Moloney murine leukemia virus (M-MLV) reverse transcriptase (Invitrogen, CA, USA) and oligo(dT). The UL15 coding sequence was amplified by PCR using the resultant cDNA and the synthetic oligonucleotide UL15f as the forward primer and UL15r as the reserve primer (Table 2). *Bam*HI and *Hin*dIII enzyme sites were incorporated into the forward and reverse primer, respectively. In order to verify the splice junction of the UL15 transcript, primers Psjf and Psjr (Table 2) were used to amplify the splice junction from the cDNA. Psjf is homologous to a sequence from exon I, 428 bp upstream from the predicted splice junction and Psjr is antisense to a sequence from exon II, located at a distance of 358 bp from the predicted splice junction. The resultant RT-PCR product was then cloned into the pMD18-T cloning vector (TaKaRa, Dalian, China) and confirmed by sequencing. For kinetic class analysis of UL15 gene, 100 μg/ml cycloheximide (CHX; Sigma-Aldrich, MA, USA) or 300 μg/ml phosphonoacetic acid (PAA; Sigma-Aldrich, MA, USA) was added to the medium 1 h before infection and maintained at this level for 6 h and 12 h, respectively. Samples with no metabolic inhibitor served as the control. RT-PCR was then carried out with the same primers as those used for the splice junction analysis with chicken β-actin mRNA as the internal control.

**Table 2:** Primers used in the this study

Primer name	**Primer sequences (5**' **to 3**'**)**	Note
P15f	ATAGGATCCATGTTCGGGGCAACTTTCG	UL15 forward/*Bam*HI^a^
P15r	GGCAAGCTTTGGTACATATCCTACGTGCGC	UL15 reverse/*Hin*dIII^a^
Psjf	TTGAATTATTCCAGAAGATGA	Splice junction forward
Psjr	TTTCTTGCATAAATGAATCG	Splice junction reverse
P898f	AATTTGGGCAACTCAATGGTT	Northern probe forward
P898r	TCTGCCGTACGTCTCATAGC	Northern probe reverse
P15-5	TGGTGCATGCTCGCAACAGT	UL15 5'-RACE primer
P15.5-5	TGTTCCAACCCGTATATTAC	UL15.5'-RACE primer
P15-3	TTACCACTGCGTGCCTCCCA	UL15 3'-RACE primer
Paf	GCGCTCGTTGTTGACA	β-actin forward
Par	TCATCCCAGTTGGTGACA	β-actin reverse

^a^The incorporated *Bam*HI and *Hin*dIII enzyme sites within P15f and P15r are underlined within primer sequences respectively.

### Amino acid sequence alignment and phylogeny

Amino acid sequences of terminase orthologues from herpesviruses and bacteriophages were retrieved from the UniProt Knowledgebase (UniProtKB) within the ExPASy proteomics server (http://www.expasy.ch/). Sequences were aligned using the ClastalW2 multiple sequence alignment program with the GONNET 250 scoring matrix [42]. The sequences are as follows: DEV (UniProtKB accession code C6ZD26), HSV-1(P04295), HCMV (P16732), Epstein-Barr virus (EBV, P03219), bacteriophage T4 (P17312), bacteriophage P7 (Q5XLR0) and bacteriophage HK97 (Q9MCT1).

For phylogenetic characterization of DEV within *Herpesviridae *with respect to the UL15 protein, a maximum likelihood (ML) phylogenetic tree based on the amino acid sequences of 23 deduced UL15 orthologues (Table 3) was constructed. Briefly, A PHYLIP format-output alignment was generated with the ClustalW2 program, with which a phylogram was computed and constructed using PhyML (version 3.0) software with the LG amino acid substitution model. An approximate likelihood ratios test (aLRT) as well as a non-parametric bootstrap support test (100 replicates) was used for phylogenetic tree evaluation. SeaView 4 was used for displaying and editing phylogenetic trees [43].

**Table 3:** Sequences used for phylogenetic analyses of large terminase orthologs from 23 herpesviruses

Subfamily	Virus species	Natural host	Abbreviation	Length		GenBank Accession
					
				nt^a^	a.a.^b^	
unassigned	Duck enteritis virus	waterfowl	DEV	2220	739	EF524094/EF203707^c^
*Alphaherpesvirinae*	Herpes simplex virus 1	Human	HSV-1	2208	735	FJ593289
	Herpes simplex virus 2	Human	HSV-2	2205	734	Z86099
	Cercopithecine herpesvirus 16	Monkey and human	CeHV-16	2208	735	DQ149153
	Marek's disease virus 1	Chicken	MDV-1	2214	737	AF147806
	Marek's disease virus 2	Chicken	MDV-2	2247	748	AB049735
	Herpesvirus of turkey	Turkey	HVT	2217	738	AF291866
	Varicella-zoster virus	Human	VZV	2232	743	DQ674250
	Bovine herpesvirus 1	Cattle	BHV-1	2208	735	AJ004801
	Bovine herpesvirus 5	Cattle	BHV-5	2214	737	AY261359
	Pseudorabies virus	Swine	PRV	2208	735	AY189899
	Equine herpesvirus 1	Horse	EHV-1	2205	734	AY665713
	Equine herpesvirus 4	Horse	EHV-4	2205	734	AF030027
	Infectious laryngotracheitis virus	Chicken	ILTV	2295	764	NC_006623
	Psittacid herpesvirus 1	Parrots	PsHV	2388	795	NC_005264
*Betaherpesvirinae*	Murine cytomegalovirus	Mouse	MCMV	2013	670	U68299
	Rat cytomegalovirus	Rat	RCMV	2013	670	AF232689
	Human herpesvirus 6	Human	HHV-6	2001	666	AF157706
*Gammaherpesvirinae*	Epstein-Barr virus	Human	EBV	2073	690	NC_007605
	Rhesus lymphocryptovirus	Monkey	RhLCV	2073	690	AY037858
	Human herpesvirus 8	Huamn	HHV-4	2073	690	NC_007605
	Murid herpesvirus 4	Mouse	MuHV-4	2040	679	AF105037
	Bovine herpesvirus 4	Cattle	BoHV-4	2049	682	NC_002665

^a^Nucleotides.

^b^Amino acids.

^c ^Fragments containing exon I and exon II were deposited separately with Accession No. of EF524094 and EF203707 respectively.

### Northen blot

Approximately 20 μg of total RNA extracted from DEV-infected CEF cells was fractionated by electrophoresis in a 1.2% agarose gel and transferred onto a positively charged nylon membrane (Immobilon™-Ny+, Millipore, MA, USA) by capillary blotting. A DIG High Prime DNA Labeling and Detection Starter Kit I (Roche Applied Science, Mannheim, Germany) was used for probe labeling and northern blot hybridization according to the manufacturer's instructions. Briefly, an 898 bp DNA fragment covering the UL15 splice junction site was amplified by RT-PCR using primers P898f and P898r (Table 2). In addition, the UL15 RT-PCR product was digested *Bgl *II, generating a C-terminus of 696 bp. These DNA fragments were purified, labeled with digoxigenin (DIG)-11-dUTP and designated P^898^, and PC^696^, respectively. The blots were hybridized at 42°C for 4 h in DIG Easy Hyb buffer followed by hybridization overnight with about 100 ng/ml labeled probes. Two 5 min stringency washes with 2 × SSC, 0.1% SDS were performed at room temperature, followed by two 15 min washes in 0.5 × SSC, 0.1% SDS at 68°C. The hybridized probes were immunodetected with anti-digoxigenin-alkaline phosphatase (AP) conjugate and were then visualized with nitroblue tetrazolium/5-bromo-4-chloro-3-indolyl phosphate (NBT/BCIP). The size of the mRNA was calculated via the RNA size standard.

### Rapid amplification of cDNA ends (RACE)

The 5'-end sequences of UL15 and UL15.5 transcripts were mapped from the purified mRNA with a 5'-Full RACE Kit (TaKaRa, Dalian, China). Briefly, total RNA was isolated from DEV-infected CEF cells with an E.Z.N.A™ Mag-Bind mRNA Kit (Omega Bio-tek, GA, USA), followed by mRNA purification using the Mag-Bind oligo(dT) magnetic beads supplied with the kit. About 250 ng of the purified mRNA was treated with calf intestinal alkaline phosphatase (CIAP) and tobacco acid pyrophosphatase (TAP) followed by ligation with the 5' RACE adaptor. RT-PCR was performed with the treated RNA using the 5' RACE primer and gene-specific primers P15-5 for UL15 and P15.5-5 for UL15.5 (Table 2). The polyadenylation cleavage site of the transcripts was determined with a 3'-Full RACE Kit (TaKaRa, Dalian, China). RT was done on the total RNA with the 3' RACE adaptor prior to the PCR reaction with the 3' RACE Outer Primer and gene-specific primer P15-3 (Table 2). Occasionally, nested PCR was used for poorly amplified products following the manufacturer's recommendation. The RACE PCR products were cloned into the pMD18-T vector (TaKaRa, Dalian, China), and the inserts were analyzed by sequencing.

### Antibodies

To produce UL15-specific antiserum, a *Bgl *II and *Hin*d III digested encoding the C-terminal 232 amino acids of UL15 exon II was inserted into the corresponding sites downstream from the T7 promotor of the pET-30a vector (Merck KGaA, Darmstadt, Germany). The recombinant plasmid, designated pET-UL15C^232^, was transformed into *Escherichia coli *competent cells BL21 (DE3). His_6_-tagged protein was expressed induced with 0.6 mM isopropyl-β-d-thiogalactopyranoside (IPTG). The cells were then harvested by centrifugation for 15 min at 3,000 × *g*. The pellet was solubilized, followed by binding to Ni-nitrilotriacetic acid agarose beads (Invitrogen, CA, USA), extensive washing and elution, and the eluted protein was renatured by dialysis. Finally, two male New Zealand white rabbits (Laboratory Animals Center, Harbin Veterinary Research Institute, Harbin, China) were immunized intramuscularly with the purified protein. Serum samples were collected when the ELISA titer reached 1:10^4^. Mouse monoclonal antibody (McAb) 1B5, raised against DEV UL19 (VP5), was prepared in our laboratory previously. Mouse anti-β actin antibody was purchased from Sigma-Aldrich Co. (St. Louis, MO, USA).

### Plasmid construction and transfection

The intronless UL15 RT-PCR fragment carrying *Bam*H I and *Hin*d III sites was cloned into the pET-30a vector (Merck KGaA, Germany), giving plasmid pET-UL15. *Bam*H I/*Not *I double digestion of pET-UL15 released the full-length UL15 ORF, which was cloned directionally into plasmid pcDNA3.1 (+) (Invitrogen, CA, USA) under the control of the human cytomegalovirus (CMV) promoter, generating plasmid pcDNA-UL15. Transient expression of the UL15 gene product was achieved in CEF cells transfected with pcDNA-UL15. Briefly, cells that grew on a coverslip placed into a 6-well culture plate (Corning, NY, USA) were transfected with 0.5 μg of plasmid using the Effectene Transfection Reagent (Qiagen, Hilden, Germany). UL15 expression was analyzed 24-48 h post transfection (h p. t.) with western blot and indirect immunofluorescence assay.

### Western blot

CEF cells were infected with DEV clone-03 in the presence or in the absence of CHX or PAA. Otherwise, CEF cells were transfected with 0.5 μg of pcDNA-UL15 or pcDNA3.1 (+) plasmid in 6-well cell culture plates. At different time points, cells were washed twice with PBS then treated with cell lysis buffer (50 mM Tris-HCl pH 8.0, 150 mM NaCl, 1% Triton X-100, 100 μg/ml phenylmethanesulfonyl fluoride) for 20 min. After centrifugation at 4°C, cell lysates were mixed with 5 × SDS sample buffer (250 mM Tris-HCl pH 6.8, 10% SDS, 5% β-mercaptoethanol, 50% glycerol, 0.05% bromophenol blue), boiled for 5 min and then analyzed by SDS-polyacrylamide gel electrophoresis (PAGE). Fractionated proteins were electrotransferred onto a nitrocellulose membrane (Millipore, MA, USA), after which the membrane was incubated in blocking solution (0.1% Tween-20 and 5% nonfat dry milk in PBS) at 4°C overnight, followed by reaction for 1 h at room temperature with the antibodies in appropriate dilution (1:200 for anti-UL15, 1:5,000 for anti-β actin), washed extensively in PBST (0.1% Tween-20 in PBS) followed by reaction for 2 h with 1:5000 diluted horseradish peroxidase-conjugated immunoglobulin (Sigma-Aldrich, MA, USA). After several washes in PBST, the bands were visualized by incubating the membrane in freshly prepared DAB solution (2.5 mg of 3,3-diaminobenzidine tetrahydrochloride in 10 ml of Tris-HCl buffer, pH 7.5) containing 0.03% H_2_O_2_.

### Immunofluorescence

Secondary CEF cells were grown on Fluorodishes (WPI, FL, USA.) and were either infected with DEV Clone-03 or transfected with 0.5 μg of pcDNA-UL15. At different time point post-infection, cells were fixed with 4% paraformaldehyde (Sigma-Aldrich, MA, USA) for 20 min at room temperature, followed by permeabilizing with 0.5% Trixon X-100 for 10 min. For cells transfected with plasmids, this procedure was done at 24 h or 48 h post-transfection. After brief washing, cells were blocked in 10% horse serum to avoid high background levels, and then incubated in 1:400 diluted UL15-specific antiserum overnight at 4°C. The cells were rinsed extensively, and then reacted with fluorescein isothiocyanate (FITC)-conjugated goat anti-rabbit immunoglobulin (Sigma-Aldrich, MA, USA) diluted 1:80. Nuclei were counter-stained with 4'-6-diamidino-2-phenylindole (DAPI; Sigma-Aldrich, MA, USA) as recommended by the manufacturer. Laser scanning confocal microscopy was performed with a Leica TCS SP5 microsystem (Leica, Mannheim, Germany).

## Competing interests

The authors declare that they have no competing interests.

## Authors' contributions

HZ and XK designed the research; HZ, HL, ZH, YS and YW performed research; HZ, HL and YW analyzed data; HZ and XK contributed to drafting the manuscript. All authors read and approved the final manuscript.
